# Historical Changes and Description of the Current Hungarian Hucul Horse Population

**DOI:** 10.3390/ani10071242

**Published:** 2020-07-21

**Authors:** János Posta, Enikő Somogyvári, Sándor Mihók

**Affiliations:** 1 Department of Animal Science, Institute of Animal Science, Biotechnology and Nature Conservation, Faculty of Agricultural and Food Sciences and Environmental Management, University of Debrecen, 4032 Debrecen, Hungary; somogyvari.eniko@agr.unideb.hu (E.S.); mihok@agr.unideb.hu (S.M.); 2 Association of Pony and Small Horse Breeders, 4032 Debrecen, Hungary

**Keywords:** Hungarian Hucul horse, pedigree structure, inbreeding, genetic variability

## Abstract

**Simple Summary:**

Originally, the Hucul horse breed was bred in the northeastern parts of the forested Carpathians. Only a few animals survived the Second World War and the regeneration of the breed started in those times. The aim of the current work was to give an overview of this rescue work from gene conservation point of view with the evaluation of the population changes within this few decades-long time interval. The pedigree quality, gene origin, inbreeding and status of stallion lines and mare families were evaluated. The main finding of the study was that inbreeding in the recent years was successfully limited and current inbreeding levels are the reason of previous gene fixations. Due to the increased number of mare families, genetic variability also increased. However, the proper management of the stallion utilization is important to prevent the future increasing of the inbreeding level of the Hucul breed.

**Abstract:**

Gene conservation and management of small populations requires proper knowledge of the background and history of the breed. The aim of the study was the evaluation of population structure and changes of the Hungarian Hucul horse population. Population changes were described for the actual breeding stock as well as for groups of 10-year epochs reflecting major periods of change in the breed. Pedigree data of the registered population were analyzed using Endog and GRain software. The average value of equivalent complete generations was above nine for the actual breeding population. The longest generation interval was the sire-to-daughter pathway. The f_e_/f ratio had smaller changes than f_a_/f_e_ ratio across the population history. Inbreeding and average relatedness as well as ancestral coefficients had increased during history. Kalinowski’s decomposition of inbreeding showed that present inbreeding is smaller than it was done earlier during the last 20 years. Due to the continuous imports from other breeder countries, the genetic variability increased during the evaluated time periods.

## 1. Introduction

The Hucul horse breed was originally formed at the borders of Bukovina, Galicia and Hungary [1]. After the Second World War, the Hungarian horse population crashed dramatically, only a few animals survived. These Hucul horses spread across the country and most of them were lost from breeding. Only a few mares were rescued by the former head of Budapest Zoo and few stallions were imported from Slovakia to start again slowly the breeding program. Two of these mares (Aspiráns (b. 1959) and Árvácska (b. 1957)) became founder of mare families. From the initial few mares and stallions, nowadays there are above greater than 300 broodmares in the actual registered breeding population and all seven recognized stallion lines are presented having more than 30 breeding stallions in Hungary. Larger and genetically important populations of the breed can be also found in Poland, Romania and Slovakia. The coordination of the across country breeding is managed by the Hucul International Federation. The total population is around 5000 broodmares presently. Following recommendations of the Food and Agriculture Organization (FAO), the Hucul breed is classified as vanishing breed and needs a conservative breeding program [1]. The genetic structure and diversity of local present populations were evaluated for the Slovak [2], the Polish [3] and the Hungarian [4] populations, but changes across birth-year time periods were not studied yet.

This breed was used as a draught horses in the past. Nowadays, Hucul horses are popular in riding schools and could be also used as leisure horses as well.

Avoidance from extreme use of stallion lines and/or mare families has special importance to maintain genetic variance and also protect stallion lines as well as mare families during gene preservation [5]. Maintaining of inbreeding and genetic diversity has special importance during gene preservation. Despite the limited population size, maintaining inbreeding at an acceptable level can be done for local populations [6,7] as well as for an international breed [8]. Careful breeding management and mating schemes are important not only for horse breeding, but other species as well [9].

Though increasing of the number of animals in the local breeding stock could be managed with import from other breeding countries, closed stud books and small population sizes require proper breeding management and mating plans to prevent high increasing of inbreeding. Gene conservation and management of endangered breeds requires proper knowledge of the genetic background and population history of the breed. The evaluation of the genealogical data of the recent decades could help to develop further breeding strategies of the breeding association. The objective of the study was the evaluation of population structure and changes over time of the Hungarian Hucul horse population based on pedigree information. An additional aim was to evaluate the changes and results of the Hungarian breeding strategy following the bottleneck after the 2nd World War. Our study focused on the evaluation of pedigree quality, gene origin, inbreeding and discover possible time of gene fixations of the actual Hungarian breeding stock and previous birth-year groups.

## 2. Materials and Methods

The stud book data of the registered Hungarian Hucul horse population up to 2019 were analyzed. The following information was stored for each animal in the database: name of the individual, male parent, female parent, birth date, sex, stallion line and mare family. The genealogical information was traced back from present horses back to the founder animals, some of them were born in the 1870s. The built database consists of the pedigree information of 10,193 horses. The changes of the population over time were evaluated from two viewpoints. First, the current breeding stock was chosen as reference population. As a second step, the Hucul horses being registered as breeding animals in Hungary were grouped into 10-year epochs reflecting major periods of change in the breed. The basis of the 10 years birth-year intervals was the average generation interval of the breed. The created intervals were preservation of the breed up to the formation of the civil breeder association (1989), expansion of the breed (1990–1999), consolidation of breeding (2000–2009), recent breeding activities (2010–2019) and current population.

The pedigree analysis was carried out using Endog [10] software. The following numbers were used to describe the different subpopulations of animals defined before:

- Pedigree completeness
  o the maximum number of generations (the number of generations separating the individual from its furthest ancestor)
  o number of full generations traced (the furthest generation where the all ancestors of the individual are known)
  o equivalent complete generations (computed as the sum over all known ancestors of the terms computed as the sum of (1 / 2)n where n is the number of generations separating the individual to each known ancestor) [11]
- Generation interval (average age of parents at the birth of their progeny kept for reproduction) [12]
- Number of founders (f: number of ancestors with two unknown parents)
- Effective number of founders (f_e_: the number of equally contributing founders that would be expected to produce the same genetic diversity as in the population under study) [13]
- Effective number of ancestors (f_a_: the marginal contributions of ancestors that would be expected to produce the same genetic diversity as in the population under study) [13]
- Number of ancestors responsible for 50% of the genetic variability (f_a_50)
- Founder genome equivalent (f_g_: Ballou and Lacy [14] defines as the number of founders that would be expected to produce the same genetic diversity as in the population under study if the founders were equally represented and no loss of alleles occurred. The parameter ƒg was obtained by the inverse of twice the average coancestry of the individuals included in a predefined reference population. [15])
- Genetic conservation index (GCI = $1\;/\;\sum p_{i}^{2}$ where p_i_ is the proportion of genes of founder i in the pedigree of an animal) [16]
- Inbreeding coefficient (the probability that the two alleles at any locus in an individual are identical by descent. The formula used for the computation is F_X_ = Σ(1 / 2)^n + n’ + 1^ × (1 + F_A_), where A is the common ancestor in the chains of origin of the father and mother of the individual X, n and n’ are the number of generations between the individual X and the common ancestor A on the paternal side (n) and the maternal side (n’), and F_A_ is the inbreeding coefficient of the common ancestor.) [17]
- Average relatedness (the probability that an allele randomly chosen from the whole population belongs to a given animal. It could be calculated using the formula: $c^{\prime }=\left(1\;/\;n\right)l^{\prime }A$, where c’ is row vector where c_i_ is the average of the coefficients in the row of individual i in the numerator relationship matrix, A, of the dimension n and A is relationship matrix of size *n* × *n*) [18]

Effective population size (the number of breeding animals that would lead to the same increase in inbreeding, as observed in the population under study, if they would contribute equally to the next generation) based on individual increase on inbreeding [5], increase in coancestry [19], log regression on equivalent generations [20] and regression on equivalent generations [21]. Ancestral inbreeding coefficients and ancestral history coefficient (A_HC_) described by Ballou et al., [22], Kalinowski et al. [23] and Baumung et al. [24] were estimated using Grain 2.2 [25] software to show the differences between time periods in inbreeding. The Hungarian Hucul horse population is quite small, so mating of related individuals could not be avoided. The idea behind computing ancestral inbreeding coefficients was to discover that alleles identical by descent for first time or were already homozygous. The following ancestral inbreeding coefficients were calculated:

- Ancestral inbreeding coefficient according to Ballou is the probability that any allele in an individual has been homozygous in previous generations at least once (F__BAL)_ [22]
- Ancestral inbreeding coefficient according to Kalinowski et al. The F__KAL_ represents that part of the genome where alleles are currently in identical by descent status and have also been identical by descent in an ancestor of the animal at least once [23]
- Proportion of alleles identical by descent for the first time are computed as F__KAL_NEW_ = F − F__KAL_, where F is the Wright’s inbreeding coefficient and F__KAL_ is Kalinowski et al.’s [23] ancestral inbreeding coefficient
- Ancestral history coefficient (A_HC_) is defined as the number that tells how many times during pedigree segregation (gene dropping) a randomly taken allele has been in identical by descent status [24].

## 3. Results

### 3.1. Quality of the Pedigree

**Figure 1:**
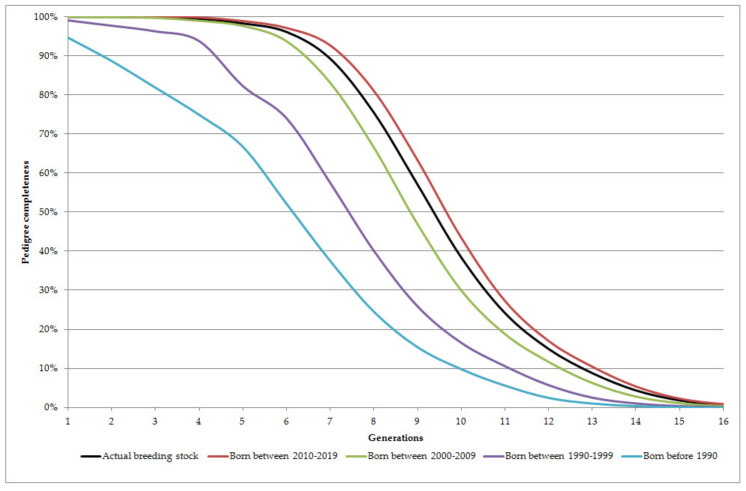
Pedigree completeness of the different reference groups.

Figure 1 and Table 1 show some pedigree completeness information of the actual breeding stock and the different birth-year groups. As most of the horses within the actual breeding stock were born in the last two birth-year groups (after 2000), average parameters of the actual breeding stock are between those of computed for the last two birth-year groups. Six generations were known completely from the pedigree information of 70% of horses born after 1990 whereas it was above 90% of horses born after 2000.

Maximum number of generations traced back to the founders was 23 for the actual breeding stock and the average was 19.56. For animals born after 2010, the mean maximum number of generations exceeded 20. The average value of equivalent complete generations is above 9 actual breeding population. The average equivalent complete generation was 5 for all subpopulations, so results of the following analyses could be handled as precise estimations for these groups [26].

### 3.2. Generation Intervals

To ensure sufficient number of parent–offspring lineages, generation intervals computed for the total population and the actual breeding stock (Table 2). The sire to offspring pathways were longer for the total population and the actual breeding stock, respectively. The longest generation interval was the sire-to-daughter pathway for the total population as well as for the actual breeding stock. The shortest pathways were the dam-to-son and dam-to-daughter pathways for the total population and the dam-to-daughter for the actual breeding stock.

### 3.3. Probability of Gene Origin

Trends in the probability of gene origin f_e_, f_a_50, and their ratios for groups based on birth years are presented in Table 3. The number of founders was higher for actual breeding stock and the later three birth-year interval groups after 1990 than the previous interval whereas half of the genetic variability was maintained with the same number of horses across different groups. The f_g_ was below 6 for the actual breeding stock and birth-year groups of horses born after 2000. The value was around 7 for birth-year groups of horses born before 1999. The f_a_/f_e_ ratio smaller than 1 (with other words: f_e_ higher than f_a_), so bottleneck effect was continuously present in the population. The f_g_/f_e_ values also show the occurrence of genetic drift for the different subpopulations. GCI was increased across birth-year groups; it is 15.1 for the actual breeding stock. The number of imported horses has been increased after the formation of breeding association and appearance of civil breeders (after 1990).

### 3.4. Inbreeding Level, Average Relatedness and Effective Population Size

**Figure 2:**
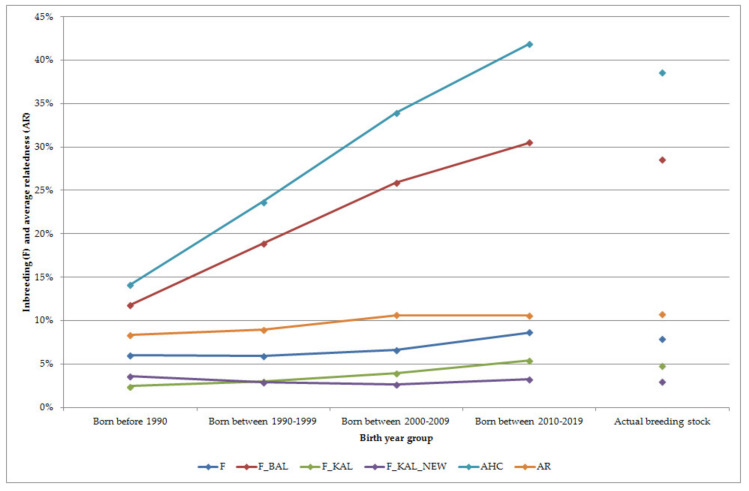
Inbreeding and average relatedness of the different groups; F—inbreeding coefficient; F__BAL_—Ballou’s formula for ancestral inbreeding; F__KAL_—identical alleles were inbred in the past; F__KAL_NEW_—identical alleles were inbred in recent generations; A_HC_—ancestral history coefficient; AR—average relatedness.

The status of the inbreeding level as well as average relatedness was estimated for each group as shown in Figure 2. Inbreeding changed near 7.5% during 50 years of breeding. Ancestral inbreeding coefficients were added to determine if inbreeding was happened in the past or in recent times. There were more differences among birth-year groups.

As it may be expected, ancestral inbreeding coefficient based on Ballou’s formula showed a higher probability of inheriting an allele that had undergone inbreeding in the past at least once for recent animals than those of within earlier born birth-year groups. The Ballou’s ancestral inbreeding coefficient and the ancestral history coefficient was higher than other estimated parameters. The ancestral history coefficient was also smaller for earlier birth-year groups and increased continuously up to recent animals, so gene dropping a randomly taken allele has been in identical by descent increased across subpopulations. The proportion of each horse’s genome that was identical by descent in an ancestor to alleles identical by descent for the first time in that horse’s lineage was estimated by the gene dropping method. The F__KAL_ increased up to recent times for birth-year groups whereas F__KAL_NEW_ was around 3% for the actual breeding stock as well as for the birth-year groups. Highest value of F__KAL_NEW_ was estimated for horses born before 1990, so some genes became identical by descent for the first time in that birth-year period. Reasons for decreasing could be the result of progenies of imported horses. The average relatedness increased over time: it was 8.36 at the beginning as the evaluation and 10.70 for the actual breeding stock.

The correlations among the inbreeding coefficients and average relatedness were also computed (Table 4.) All estimated values were significant (*p* < 0.01). As there is a relationship in the computation of F__KAL_ and F__KAL_NEW_ to the classical inbreeding coefficient (F), strong correlations were estimated. Ballou’s ancestral inbreeding coefficient was in very strong correlation with the ancestral history coefficient and in strong correlation with F__KAL_. In addition, strong correlation was estimated between F__KAL_ and the ancestral history coefficient.

The effective population size was estimated in different ways (Table 5). The effective population size based on inbreeding (Ne_F_ and Ne_Coan_) was a little higher than 50 for birth-year groups 1990–1999 and 2000–2009. It may be due to the intensive import of breeding animals from other breeding countries. Nowadays, it was estimated below 50 for the actual breeding stock, and for animal born between 2010–2019. The regression-based effective population sizes (Ne_log_ and Ne_reg_) were quite close to each other during population history and were around 20.

## 4. Discussion

The maximum number of generations traced and average equivalent complete generations computed for birth-year groups after 2000—as well as for the actual breeding stock—were higher than [2] estimates for the Slovakian Hucul horse population and [3] reported for the Polish Hucul horses. Pedigree completeness data also suggest more deep pedigree information for the actual Hungarian breeding stock, as well as for animals born after 1980. Recently computed numbers are slightly higher than those reported by [4]. Recent equivalent complete generations are higher than was found for the Asturcón pony population [7].

The longest generation intervals estimated for the sire pathways may be the reason of the selection method of breeding stallion that resulted in receiving breeding license in later age. Mares are usually starting their breeding career at three years and having first foal at four years of age. Our findings for sire pathways are close to estimations for the Slovakian Hucul population [2] whereas dam pathways are shorter than that value. The tendency of the computed values in the recent study was quite similar to those of reported by [4]. Our estimations were longer than found for different Noriker populations [27,28].

Effective number of founders and effective number of ancestors reached the top in the Hungarian population for breeding animals born between 1990 and 1999. Our estimations are quite similar to those of estimated some years before [4]. These estimated numbers are in alignment with findings for the Slovakian Hucul horses [2]. The number of ancestors responsible for 50% of the genetic variability was also similar to their findings in our recent groups. As the Hucul horse has closed pedigree, these similar numbers may strengthen that there is reasonable relationship between the breeding stocks of the two countries. The estimated f_a_ values are in agreement with those of reported for Spanish Arab horse [29] and Asturcón pony [7], but smaller than the internationally bred Lipizzan horse [8]. This suggests that small populations, which are bred only in few countries and having closed pedigree, are more endangered than worldwide known breeds. The f_e_ values are quite similar to estimates for the Asturcón pony [7], but much smaller than Arabian [29], Andalusian [30] and Lipizzan horses [7]. Though the use of breeding animals was not completely balanced in the population under study, the f_a_ and f_e_ values are much favorable than reported for the Sorraia breed [31]. Genetic loss during the monitoring of the Hucul Hungarian breeding stock based on the f_a_/f_e_ ratio is similar those of obtained for Asturcón pony [7]. The reorganization of civil breeding activities was allowed, and the main priority of the breeder association was to increase number of individual of the population. This may be the reason of the change in the number of imported animals from the birth period 1990–1999. As the present population size is above 300 broodmares, this tendency is slowing and focuses only to increase genetic variability of the breed, mainly with importing mares belonging to mare families not yet present in Hungary.

Compared to [4], we estimated higher inbreeding coefficients, so the breeder association should pay attention to more careful mating plans during their work. Inbreeding coefficients increased only slightly across birth-year groups. Increasing in inbreeding coefficient is in alignment with [32] results for Asturcón pony breed. Wright’s coefficient as well as the average relatedness is quite close to [2] estimations for the Slovakian Hucul population and in agreement with [3] calculated for Polish Hucul horses. The probability that an allele has been homozygous in previous generations was in above 20% for subpopulations born between 2000–2009 and 2010–2019 as well as for the actual breeding stock. This parameter is not available for other horse breeds yet and smaller those of reported for Border Collie dogs [3]. Kalinowski’s new formula also showed that new fixation of genes increased in this period. Kalinowski’s and Kalinowski’s new formula were not used for horses before, the fixation tendency based on these parameters is not in agreement with that of reported for the Hungarian Border Collie population [9]. Due to the political changes in the Eastern part of Europe, the exchange of breeding animals across countries became easier and this result a low decrease for these parameters in case of birth-year groups 1990–1999 and 2000–2009. The appearance of encouraged private breeders also made the genetic variability wider and made it easier to avoid from the mating of close relatives. Due to the closed pedigree of the breed, within the last decade almost all parameters increased, so the breeder association must be more careful and should be take into account the F and AR values during preparation of mating plans for upcoming years. Inbreeding of the actual breeding stock and recently born horses is in alignment with values obtained for Spanish Arabian [29] and Andalusian [30] horses. Zechner and colleagues [8] reported higher values for Lipizzan horses whereas [7] found lower inbreeding for Asturcón pony than our estimations. AR values of the recent groups of Hucul horses are in agreement with the estimations of Spanish Arabian [29], Asturcón pony [7] and Andalusian [30] horses. Our estimated AR values were higher than half of the F for the actual population as well as all birth-year groups, so mating of relatives could not be avoided in the Hungarian Hucul breeding stock during its history. The ancestral history coefficient has been only rarely studied yet. Our estimated values were higher for all birth-year groups than published for Angler and Red-and-White cattle breeds [33]. The tendencies of the estimated correlations of the different inbreeding parameters in our study were similar to those of reported for Angler and Red-and-White cattle [33].

The estimated effective population size for the actual breeding stock and animals born between 2010–2019 show narrowing in the genetic diversity and as it decreased below 50, it suggest problem with the sustainability of the population [34]. Furthermore, log and linear regression give very similar results, this is because in most of datasets the range of inbreeding coefficients is so narrow that a linear approximation gives basically the same result. It is in agreement with [2] reported for Slovakian Hucul horses and [3] for the Polish Hucul horse population. These similar values could also confirm similarity in breeding management of Hucul horse populations. Our estimations are quite similar to those for Silesian Noriker horses [28], but smaller than estimates for the internationally bred Lipizzan horses [8]. The effective population size based on inbreeding and on coancestry was quite similar during the population history. This suggests that there is intensive exchange between breeding stocks and there are no subpopulations among breeders/breeder countries. The ratio of Ne_Coan_ and Ne_F_ also suggests that there are no genetic lines in the population, we can talk about only genealogical lines. Estimations based on regression are smaller than those of based on inbreeding and coancestry which is in agreement with results reported for several Spanish horse populations [35]. Leroy and colleagues [36] reported about the comparison of different methods of estimation of effective population size. They made a great emphasize on the effect of pedigree completeness on the estimated effective population size. Average equivalent complete generations were above five for each evaluated subpopulations, so the estimations based on the inbreeding are reliable. To monitor population changes, we should pay attention to methods which can respond quickly to changes in the population. The regression-based computation methods use the shortest time window for the estimations. In such cases we can use regression-based effective population sizes which both below 20 for recent subpopulations and suggest that variability is decreasing in the Hungarian Hucul population.

Careful mating management is required, as the Hungarian population is endangered based on the effective population size. More equal distribution of stallions across lines may be favorable. The diversity of mare families—as well as the size of the different families—should be more balanced. These activities could help the maintenance and preservation of lines and families, as well as the diversity of the breed.

## 5. Conclusions

Increasing of the breeding population of Hucul horses was successful without any reasonable gene losses and changing in inbreeding level. The average value of equivalent complete generations was above nine for the actual breeding population. The longest generation interval was the sire-to-daughter pathway, whereas it was the shortest for the dam-to-son and dam-to-daughter pathways. The possibility of moving of breeding stock in the last decades increased frequency of identical alleles that were inbred in the past, while recent fixation of alleles have not changed. Effective population size showed that Hungarian Hucul population is endangered, and sustainability requires proper breeding management.

## Figures and Tables

**Table 1:** Description of the quality of the pedigree.

Population	Number of Animals	Average of Maximum Number of Generations	Average Number of Full Generations Traced	Average Equivalent Complete Generations
Actual breeding stock	420	19.56	5.30	9.10
Born between 2010–2019	572	20.04	5.45	9.41
Born between 2000–2009	574	19.00	4.98	8.58
Born between 1990–1999	166	17.23	3.89	7.04
Born before 1990	46	14.85	2.76	5.56

**Table 2:** Descriptive statistics of the generation intervals in years.

Pathway	Generation Interval (Standard Error)	
	Total Population	Actual Breeding Stock
Sire-to-son	11.29 (0.21)	10.22 (0.91)
Sire-to-daughter	11.44 (0.10)	12.55 (1.31)
Dam-to-son	9.28 (0.19)	11.76 (1.63)
Dam-to-daughter	9.29 (0.09)	8.99 (1.10)

**Table 3:** Demographic parameters of the Hungarian Hucul horse population by birth year.

Parameter	Actual Breeding Stock	Born between 2010–2019	Born between 2000–2009	Born between 1990–1999	Born before 1990
f	104	105	102	102	89
f_e_	22	21	24	28	23
f_a_	16	16	16	17	16
f_a_50	6	6	6	6	6
f_g_	5.78	5.48	5.84	7.03	7.51
f_e_/f ratio	0.21	0.20	0.24	0.27	0.26
f_a_/f_e_ ratio	0.73	0.76	0.67	0.61	0.70
GCI	15.1 ± 2.08	15.3 ± 1.88	15.2 ± 2.41	13.1 ± 3.58	8.9 ± 3.91
N_S_	13	3	19	16	12
N_M_	42	21	54	43	13

f—number of founders; f_e_—effective number of founders; f_a_—effective number of ancestors; f_a_50—number of ancestors responsible for 50% of the genetic variability; f_g_—founder genome equivalent; GCI—gene conservation index; N_S_—number of imported stallions; N_M_—number of imported mares.

**Table 4:** Correlation among different inbreeding coefficients and average relatedness.

Coefficients	F__BAL_	F__KAL_	F__KAL_NEW_	A_HC_	AR
F	0.495	0.860	0.905	0.479	0.504
F__BAL_		0.772	0.170	0.996	0.627
F__KAL_			0.574	0.768	0.527
F__KAL_NEW_				0.146	0.378
A_HC_					0.586

F—inbreeding coefficient; F__BAL_—Ballou’s formula for ancestral inbreeding;—identical alleles were inbred in the past; F__KAL_NEW_—identical alleles were inbred in recent generations; A_HC_—ancestral history coefficient; AR—average relatedness.

**Table 5:** Effective population sizes of the different groups.

Group	Ne_F_	Ne_Coan_	Ne_log_	Ne_reg_
Actual breeding stock	49.54	48.00	19.10	19.62
Born between 2010–2019	46.35	49.07	18.30	18.52
Born between 2000–2009	55.55	47.52	16.73	17.09
Born between 1990–1999	52.43	47.06	24.88	24.76
Born before 1990	40.93	37.67	20.68	20.80

Ne_F_—effective population size computed via individual increase in inbreeding; Ne_Coan_—effective population size computed via individual increase in coancestry; Ne_log_—effective population size obtained from log regression on equivalent generations; Ne_reg_—effective population size computed via regression on equivalent generations.
